# CoViNAR: a context-aware social media dataset for pandemic severity level prediction and analysis

**DOI:** 10.3389/frai.2025.1623090

**Published:** 2025-08-20

**Authors:** Soofi Shafiya, Mudasir Ahmad Wani, Suraiya Jabin, Mohammad ELAffendi, Jahiruddin

**Affiliations:** ^1^Department of Computer Science, Faculty of Sciences, Jamia Millia Islamia, New Delhi, India; ^2^EIAS Data Science & Blockchain Laboratory, College of Computer and Information Sciences, Prince Sultan University, Riyadh, Saudi Arabia

**Keywords:** BERTopic, COVID-19, natural language processing, social media, DistilBERT, SVM

## Abstract

**Introduction:**

The unprecedented COVID-19 pandemic exposed critical weaknesses in global health management, particularly in resource allocation and demand forecasting. This study aims to enhance pandemic preparedness by leveraging real-time social media analysis to detect and monitor resource needs.

**Methods:**

Using SnScrape, over 27.5 million tweets for the duration of November 2019 to March 2023 were collected using COVID-19-related hashtags. Tweets from April 2021, a peak pandemic period, were selected to create the CoViNAR dataset. BERTopic enabled context-aware filtering, resulting in a novel dataset of 14,000 annotated tweets categorized as “Need”, “Availability”, and “Not-relevant”. The CoViNAR dataset was used to train various machine learning classifiers, with experiments conducted using three context-aware word embedding techniques.

**Results:**

The best classifier, trained with DistilBERT embeddings, achieved an accuracy of 96.42%, 96.44% precision, 96.42% recall, and an F1-score of 96.43% on the Test dataset. Temporal analysis of classified tweets from the US, UK, and India between November 2019 and March 2023 revealed a strong correlation between “Need/Availability” tweet counts and COVID-19 case surges.

**Discussion:**

The results demonstrate the effectiveness of the proposed approach in capturing real-time indicators of resource shortages and availability. The strong correlation with case surges underscores its potential as a proactive tool for public health authorities, enabling improved resource allocation and early crisis intervention during pandemics.

## 1 Introduction

Epidemiologists aim to learn vital lessons from pandemics to build resilience and prepare better for future health emergencies. Social media platforms, especially Twitter (now X), have emerged as critical tools for real-time information dissemination, public sentiment analysis, and resource monitoring during crises (Gupta and Katarya, 2020). The COVID-19 pandemic, which originated in China in December 2019 and spread worldwide in 2020, highlights the importance of social networks for both public health agencies and the general population. Netizens around the world shared extensive information, discussing emotions, case numbers, resource needs, and symptoms. Social media platforms were flooded with intense reactions due to the COVID lockdown and unprecedented death tolls. This enabled analysis using real-time social media data, with primary applications including sentiment analysis, disease surveillance, and activity monitoring (Wang et al., 2023).

Public health agencies are increasingly relying on social media to address public concerns, promote preventive measures, and detect emerging health threats. In particular, sentiment analysis has proven to be valuable in gauging public perception and emotional responses to health-related issues (Wani et al., 2019). Studies using Natural Language Processing (NLP) techniques have examined the negative correlation between local COVID-19 severity and sentiments during lockdown (Pastor, 2020), and increased negative emotions and social threat susceptibility post-pandemic declaration (Medford et al., 2020). Early sentiment detection enables more effective understanding and management of pandemics (Jalil et al., 2022). Advances in NLP–from basic “bag-of-words” models to sophisticated frameworks like GPT and BERT–have significantly improved the accuracy and nuance of social media analysis, empowering more targeted and effective public health responses.

In addition to sentiment analysis, social media data have proven to be very helpful in predicting COVID-19 case trends (Li et al., 2020), detecting toxic fake news during COVID-19 (Wani et al., 2024), depression screening (Ahmad Wani et al., 2023), modeling the dynamics of COVID-19 (Nelson et al., 2024), etc. Geo-located data can identify hot spots with high health discussions or symptom reports, enabling targeted resource allocation and interventions. Researchers have used X data, Google search trends, and Wikipedia traffic to build a leading indicator model for predicting COVID-19 infection rates and Mortality patterns among Americans (O'Leary and Storey, 2020). Recent work by Shafiya and Jabin (2023) has further synthesized advances in social media-based surveillance systems for early outbreak prediction and monitoring.

During COVID-19, critical resource shortages led to increased discussions on social media about resource demand and availability, as illustrated in Table 1. Monitoring and quickly identifying growing resource needs is essential for timely actions to mitigate public health challenges. In response, this work introduces an innovative approach focused on filtering and classifying tweets that express either the “Need” or “Availability” of resources (NAR). We present CoViNAR, a newly labeled dataset comprising 14,000 NAR tweets, which addresses the gap in existing COVID-19 tweet corpora that lack such specialized datasets. Our methodology employs state-of-the-art NLP techniques, including BERTopic for initial data filtering and topic modeling, followed by a Machine Learning (ML) pipeline for NAR classification. Multiple contextual embedding techniques (BERT, RoBERTa, DistilBERT) paired with various classifiers are evaluated to achieve optimal performance.

**Table 1:** Examples of “need” and “available” tweets.

**Tweet**	**Category**
I need an #oxygen can in #Kolkata. Please provide me recent #Verified leads regarding the same. Please #Amplify #CovidHelp #CovidIndia #COVIDEmergency2021 #COVID19 #KolkataCovid	Need
Very urgent !!! Medical Emergency. Anyone who has an idea about the availability of this injection please dm me. Please spread the word, if possible #Surat #Surtis #Emergency #coronavirus #COVID19	Need
It's an emergency for ICU bed with Ventilator in Bangalore. Please send some verified leads. We are in search from yesterday. #bangalore #COVIDEmergency #VentilatorBeds #ICUBedSOS #covid	Need
Available: #Delhi #Oxygen refill (no cylinder) from 11 a.m. April 23. #CovidSOS #SOSDelhi #COVIDEmergency #Covid #DelhiNeedsOxygen	Available
All medical appliances available. Oxygen will be available from tomorrow (No injections). #Ahmedabad #CovidIndia #COVIDEmergency2021 #COVID19 #CovidHelp #Verified	Available
@UzmaaP #COVID #verified #Surat Plasma. Contact: 88665155368. Service Available: Plasma. City: Surat. Plasma donation and requirement help desk. Verified on: 24 April 04:41 pm.	Available

Recognizing pandemic severity is crucial for implementing timely and effective control measures. The present work explores the significance of social media posts expressing resource needs and availability on X. The tweets were classified as “Need” and “Availability” of resources (NAR). Their frequency was used to assess the increasing seriousness of COVID-19. Despite existing COVID-19 tweet corpora, labeled data classifying resource demand/availability was lacking. This gap was bridged by using topic modeling and NLP to annotate social media data, for creating the CoViNAR dataset.

Approximately, 27.5 million public tweets were scraped from X spanning November 2019 to March 2023, covering pre-pandemic, peak, and post-peak periods. From this collection, we selected a subset of 1 million tweets from April 2020 (one of the pandemic's peak months). Using topic modeling and NLP methods, irrelevant tweets were filtered out and the remaining ones were categorized, yielding a final dataset of 14,000 labeled tweets across three classes: “Need,” “Availability,” or Other (negative class). An ML-based “NAR” classifier is trained to categorize tweets into these three classes. Automatically identifying NAR tweets can help healthcare organizations map deficits and surpluses in medical supplies, hospital capacity, and other needs communicated online during health emergencies, regardless of the disease outbreak. By monitoring NAR trends on social media, public health officials can enable data-driven resource allocation to areas of greatest need. Integrating our NAR classifier with existing public health monitoring systems will significantly enhance pandemic preparedness and response capabilities for future outbreaks.

The current work aims to address the following research questions (RQ):

- RQ1. Can social media data be effectively utilized for real-time resource management during a pandemic?
- RQ2. How accurately can social media analysis reflect the real-time severity level of a pandemic?
- RQ3. Can social media analysis potentially complement or enhance traditional surveillance methods for resource allocation management?

The following are the significant contributions of the work in the light of the hypothesized research questions:

- This paper introduces a novel labeled “CoViNAR” COVID-19 X tweets dataset with 14,000 NAR tweets. The CoViNAR dataset is publicly available at GitHub.
- We implemented a state-of-the-art contextual topic modeling approach by fine-tuning BERTopic, for initial filtering and automated labeling, allowing us to efficiently identify tweets relevant to resource “needs”, “availability”, and “not-related”. This pre-filtering process minimizes information overload by excluding irrelevant content, significantly reducing the manual effort required in labeling.
- This work presents a novel framework for accurately identifying and classifying tweets in 3 categories viz. “Need” or “Availability” of resources (NAR), or unrelated class.
- Three pre-trained contextual text embeddings are used and compared to generate feature vectors for the training of the NAR classifier.
- The trained classification model achieved 96.42% accuracy on the labeled Test dataset. A temporal analysis is performed to establish a direct link between the count of classified NAR tweets and COVID-19 progression over three years from Nov 2019 to Mar 2023.

The key contributions of our work include: a tweet dataset for NAR classes, a context-aware NLP pipeline, temporal analysis of social media data, and COVID-19 severity analysis. The approaches described provide authorities with an important supplemental monitoring tool for identifying, prioritizing, and responding to developing outbreak hotspots. The rest of the content of this paper is structured as follows: Section 2 discusses related studies. Section 3 covers materials and methods. Section 4 outlines the classification model. Section 5 presents the experiment results. Section 6 provides a geographic location-specific temporal analysis of COVID-19 tweets. Section 7 concludes the work.

## 2 Related work

The COVID-19 pandemic has significantly accelerated research into using social media for public health monitoring and response, sparking extensive studies across multiple domains.

While social media was previously considered only supplementary for disease tracking (Wilson et al., 2021), it gained significant importance during the COVID-19 pandemic. Research by Syrowatka et al. (2021) identified six potential AI use cases for pandemic preparedness and response, including diagnosing influenza-like diseases, triaging infections, and evaluating prognosis. Their work highlighted AI's role in providing timely insights beyond traditional models. Research on Arabic NLP and machine learning highlights the adaptability of NLP techniques across languages, strengthening multilingual NLP and enhancing transformer-based models and topic modeling like BERTopic for large-scale text analysis (Marie-Sainte et al., 2018). Such advancements enhance content moderation, misinformation detection, and trust in online platforms.

Since COVID-19's emergence, studies have extensively analyzed social media data, especially from platforms like Facebook and X, for sentiment analysis (Kaur et al., 2021). A recent study (Da and Yang, 2020) analyzed public sentiments on social media during the COVID-19 pandemic in China using the “SKEP” model. Their approach combined advanced NLP techniques with traditional ML models like random forests and linear probit to achieve both high accuracy and interpretability. Their findings established a negative correlation between local COVID-19 severity and public sentiment, highlighting the critical role of social media in reflecting public response during health crises. Another study (Nemes and Kiss, 2021) demonstrated the superiority of RNN models over traditional methods like TextBlob for sentiment prediction, emphasizing the value of advanced NLP techniques for extracting meaningful insights from social media data during health emergencies. Further research (Melton et al., 2022) analyzed public sentiment on COVID-19 vaccines across 9.5 million tweets and 70,000 Reddit comments, using a fine-tuned DistilRoBERTa model. Their study revealed platform-specific differences: X showed more negative sentiment (54.8%) compared to Reddit's predominantly positive sentiment (62.3%). Their work highlights the importance of considering multiple social media sources when assessing public opinion during health crises.

Beyond sentiment analysis, researchers have utilized social media for forecasting COVID-19 case numbers (Lamsal et al., 2022). Study (Qin et al., 2020) demonstrated the potential of social media search indexes to predict new suspected and confirmed cases, while Li et al. (2020) found strong correlations between “coronavirus” searches and confirmed COVID-19 cases occurring 8–12 days prior by analyzing Google Trends, Baidu, and Sina Weibo data. More sophisticated modeling approaches have also emerged. Research (Verma et al., 2022) developed a CNN-LSTM model to capture the complex trend of COVID-19 outbreaks. This work predicted daily confirmed cases for India and its most affected states, showing that the stacked LSTM and hybrid CNN-LSTM models performed relatively better than others.

While these studies demonstrate the potential of social media data for pandemic monitoring and prediction, a significant gap remains in exploring X data specifically for identifying resource needs and availability during crises. Recent research has highlighted the critical importance of effective resource management during health emergencies. Kaur et al. (2024) investigated the relationship between negative sentiment in COVID-19-related tweets and intensive care unit (ICU) bed demand across regions in the United States, Brazil, and India. Their findings underscore the potential of social media sentiment analysis as an early warning system for medical resource demand surges, complementing our focus on explicitly identifying resource needs and availability through tweet content.

Other researchers have explored various aspects of pandemic resource management beyond social media analysis. Research (Borges and Nascimento, 2022) proposed a hybrid Prophet-LSTM approach for ICU demand forecasting that achieved higher accuracy than standalone models. Similarly, Goic et al. (2021) developed a hybrid forecasting model for short-term ICU utilization in Chile that was actively used in capacity planning decisions. These studies, while not directly using X data, highlight the importance of accurate resource forecasting in pandemic response.

The COVID-19 pandemic also exposed significant weaknesses in global supply chains and resource distribution. Research (Srinivas et al., 2021) examined how the pandemic revealed shortcomings in supply chain operations, particularly for essential medical goods, while proposing recommendations to improve resilience. Study (Ranney et al., 2020) addressed the critical shortage of ventilators and personal protective equipment in the United States early in the pandemic, highlighting the uncertainty in estimating resource needs. Further research (McMahon et al., 2020) emphasized the severe lack of basic medical supplies in low-income countries, underscoring global disparities in resource allocation. Another study (Zhuang et al., 2021) estimated hospital bed shortages during Wuhan's lockdown, emphasizing the importance of lockdown timing and strength in managing healthcare system capacity.

Although these studies did not utilize social media data, they provide crucial context for understanding the real-world implications of resource shortages during pandemics. There remains a considerable opportunity to utilize X data more effectively for detecting and forecasting resource needs and availability. Our work aims to address this gap by introducing the CoViNAR dataset, which specifically focuses on tweets about resource needs and availability during COVID-19. By employing advanced NLP techniques like BERTopic for data filtering and transformer-based embeddings for classification, we propose to develop models that can directly inform resource allocation efforts based on real-time social media data.

## 3 Materials and methods

This section outlines the methodology for developing a systematic approach to identify and classify COVID-19-related tweets. It describes the data sourcing process, dataset creation methods, development of the NLP pipeline, model training procedures, and performance evaluation techniques. The same pipeline can be extended for analyzing other disease outbreaks.

### 3.1 Source of dataset

The already existing publicly available COVID-19-related datasets (sourced from X/Facebook) are mainly centered around emotion analysis. Researchers have tried to correlate emotions expressed by netizens to the severity of the pandemic by analyzing tweet sentiments. However, no available dataset specifically addresses resource needs and availability during the COVID-19 pandemic.

For the current work, we collected public tweets from the social media platform “X” spanning November 2019 to March 2023, covering all pandemic stages viz. pre-pandemic, early pandemic, and peak periods. The scraping of publicly available tweets complied with the platform's terms of service until March 2023. We extracted relevant tweets using COVID-19-related hashtags including “#coronavirus, #covid, #covid-19, #pandemic, #lockdown, #virus, #outbreak, #China, #deaths.” Our scraping process collected data in 10-day intervals with a limit of 200,000 tweets per scrape, systematically capturing daily tweets across the 3-year period. After aggregating all the daily collections, we obtained ~ 27.5 million tweets from November 2019 to March 2023. Among these tweets, some included geo-location data, which were utilized for further temporal analysis of the three most affected countries: India, the United States, and the United Kingdom.

Following data collection, we preprocessed tweets to prepare them for analysis, selecting only English-language content since English was the dominant language in tweets from India, the United States, and the United Kingdom, the three countries used for temporal analysis. Pre-processing is an important step when working with unstructured data, as it eliminates noise and inconsistencies (Maharana et al., 2022). For this purpose, an open-source Python library Natural Language Toolkit (NLTK) was utilized. Preprocessing of tweets included removal of URLs, hashtags, user mentions, special characters, duplicates, and finally lowercasing the cleaned text. This preprocessing was applied uniformly to each tweet.

We selected April 2021 tweets as training data for our NAR classification model since this represented a global peak period for COVID-19. Approximately 1 million tweets were scraped for April 2021, and the preprocessing step reduced this to about 800,000 tweets. We subsequently applied this NAR model to classify tweets across our entire study period (November 2019–March 2023) for pandemic analysis, as detailed in Section 6. The following subsections describe our NLP pipeline after preprocessing.

### 3.2 Topic modeling

**Figure 1:**
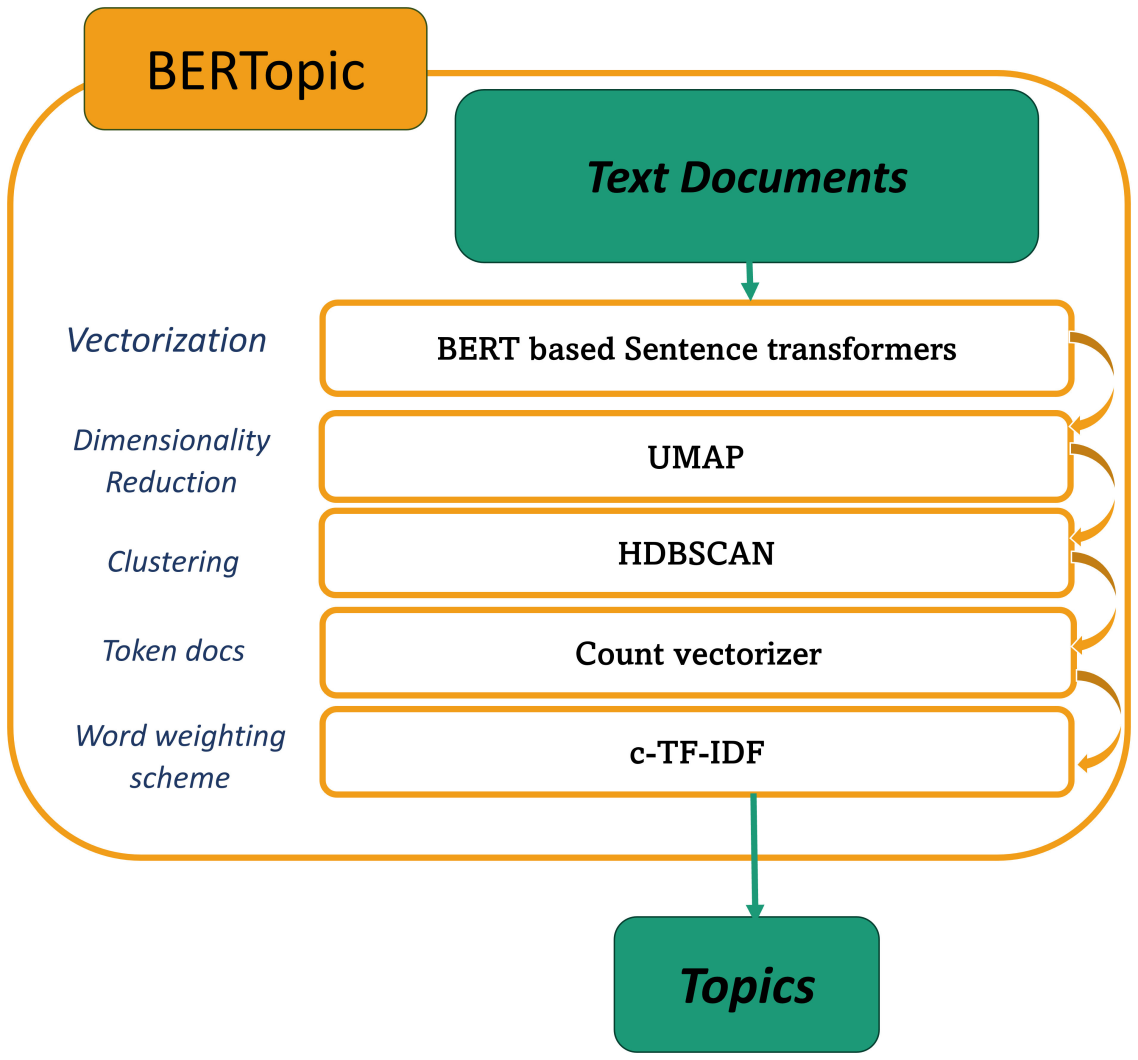
Overview of BERTopic.

Topic modeling is a significant approach in NLP for identifying hidden topics and patterns in large corpora, which facilitates tasks such as document search, categorization, summarization, and recommendation. For the proposed work, we employed topic modeling as a filtering technique to eliminate irrelevant tweets. Commonly used techniques include LDA, LSA, NMF, and BERTopic. unlike other approaches, BERTopic explores semantic depth by utilizing dense vector representations generated by pre-trained BERT models (Egger and Yu, 2022). An overview of BERTopic is illustrated in Figure 1. It entails encoding text into vectors with Sentence Transformers (Reimers and Gurevych, 2019), followed by dimensionality reduction with UMAP (McInnes et al., 2018), which maintains both local and global properties effectively.

HDBSCAN (McInnes et al., 2017), a hierarchical approach that handles changing cluster densities, is then employed for data clustering. This approach enhances topic representation quality through its noise-aware soft-clustering methodology, which explicitly identifies and excludes outliers. The dimensionality reduction performed by UMAP significantly improves both the clustering accuracy and computational efficiency compared to traditional methods like K-means (Allaoui et al., 2020). Count Vectorizer is used to create a bag-of-words representation of text data. This step is required to extract the most representative words or phrases that describe each discovered topic cluster. Finally, c-TF-IDF (Grootendorst, 2022) calculates the significance of words in each topic cluster, considering both the frequency of terms and the inverse document frequency within that specific cluster. This aids in identifying the most pertinent and discriminative words associated with each topic. All things considered, BERTopic, a state-of-the-art (SOTA) technique in this field, transforms conventional topic modeling approaches by utilizing the revolutionary potential of BERT embeddings.

### 3.3 Feature extraction

Once filtered tweets were obtained, text vectorization transformed them into numerical representations for machine processing. While non-contextual techniques like GloVe (Pennington et al., 2014) and Word2Vec (Jatnika et al., 2019) are popular, they lack contextual understanding. The proposed work utilized contextual embeddings to capture the semantic nuances of tweets more effectively. These embeddings (Roman et al., 2021) provided richer representations by considering the surrounding context of words, enhancing the classification of disease outbreak-related tweets. The details of the employed embedding models are provided below:

#### 3.3.1 BERT-base

Devlin et al. (2019) is a deep bidirectional transformer-based model trained on large-scale corpora. It learns contextualized word embeddings by considering both left and right contexts. We employed the BERT-base (110 Million (M) parameters) model to extract 768-dimensional dense vector embeddings for each tweet. Additionally, larger variants such as BERT-large (340M parameters) exist, but for computational efficiency, we used BERT-base.

#### 3.3.2 DistilBERT

A compressed variant of BERT with similar embedding dimensions but optimized for efficiency through knowledge distillation, enables it to be smaller and more efficient while retaining most of BERT's performance (Sanh et al., 2019). The model has 66M parameters, making it more efficient for large-scale tweet processing without significant performance loss. DistilBERT was used to generate 768-dimensional tweet embeddings efficiently.

#### 3.3.3 RoBERTa

An advanced version of BERT with refined contextual representations due to improved pretraining procedures on larger datasets (Liu et al., 2019). We employed the RoBERTa-base (125M parameters) variant, which provides better contextual representations compared to BERT. A larger RoBERTa-large (355M parameters) variant is also available, but due to computational constraints, we used the base version. RoBERTa was also used to generate 768-dimensional tweet embeddings.

We experimented with all of these contextual embeddings and found DistilBERT the most suitable one for the CoViNAR dataset concerning Need/Availability classification tasks.

### 3.4 Classification models

This section briefly introduces the classification algorithms used for the text classification task in the proposed work.

#### 3.4.1 Support vector machines

A supervised ML technique that identifies the optimal hyperplane in feature space to classify data points, by maximizing the margin between classes (Cortes and Vapnik, 1995).

#### 3.4.2 Random forest

An ensemble method that combines classification from multiple decision trees by bagging or boosting (Breiman, 2001). It is a robust model that trains underlying estimators by forming multiple subsets of Train data with bootstrapping.

#### 3.4.3 Logistic regression

A binary classification model that uses a sigmoid-transformed linear function, valued for its simplicity and efficiency in tasks with linear decision boundaries (Hosmer Jr et al., 2013).

### 3.5 Evaluation metrics

Model's performance was assessed through standard classification metrics including:

Accuracy, Precision, Recall, F1 score, Confusion Matrix, etc.

## 4 NAR classification model

All experiments were conducted using VSCode on a machine equipped with 64 GB RAM and an NVIDIA GPU with 16 GB memory. Standard libraries such as NLTK (Bird, 2006) and Python's re module were utilized for text pre-processing and dataset preparation, while Scikit-learn (Pedregosa et al., 2011) was used for ML classification tasks. GridSearchCV-based hyperparameter tuning and classifier evaluations were carried out using this computational setup, which ensured efficient execution of embedding generation, model training, and evaluation across multiple configurations. This section details the preparation of the CoViNAR dataset, classification model design, and hyperparameter tuning process. The current experimental framework is tailored to reliably analyze COVID-19 severity by analyzing the frequency of NAR tweets.

### 4.1 Dataset preparation

**Figure 2:**
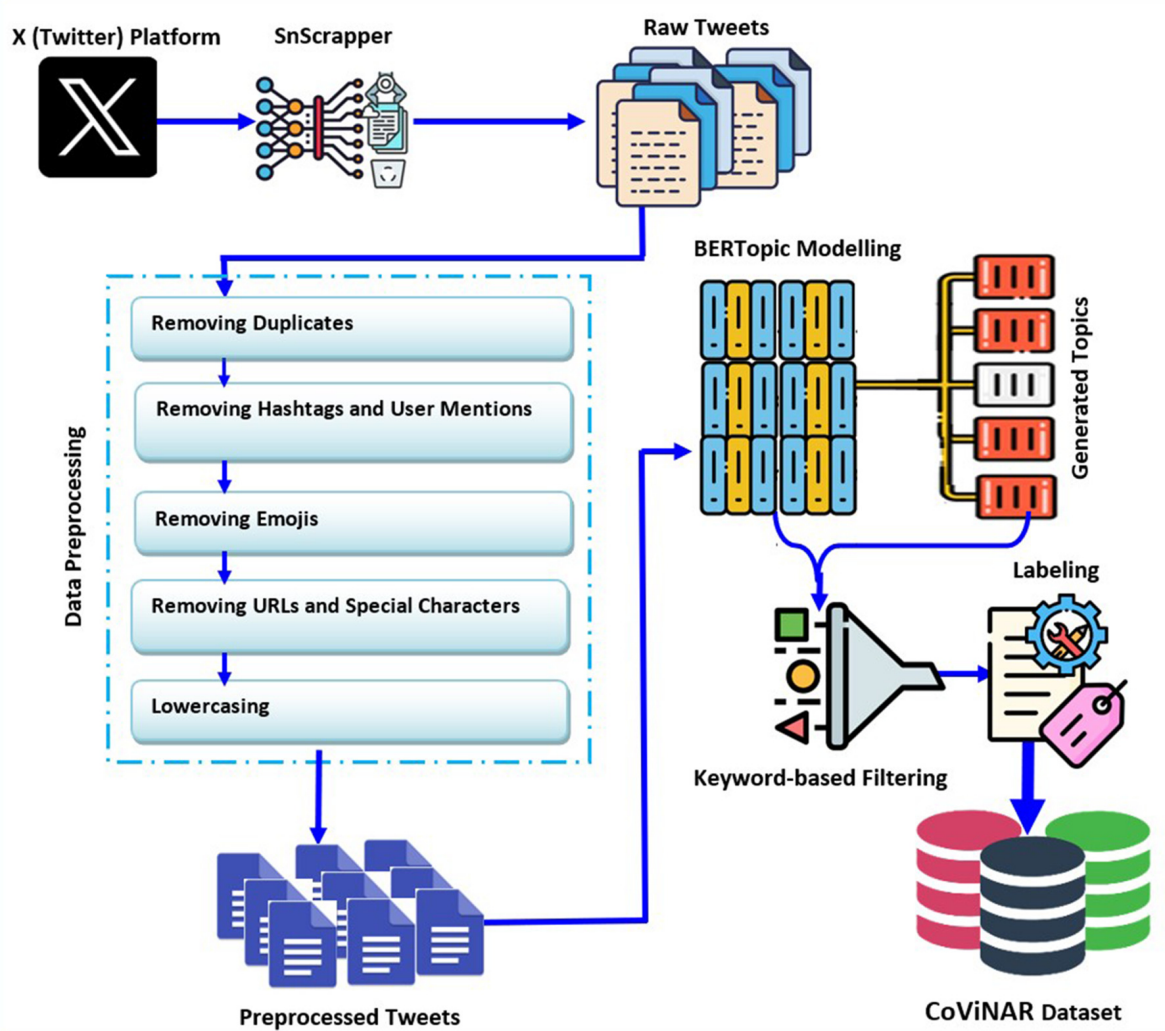
Methodology for constructing the CoViNAR dataset.

This subsection outlines the dataset preparation, covering data pre-processing, filtering, and tweet labeling sequentially, as depicted in Figure 2. The overall workflow is also presented in Algorithm 1, which details the key steps involved in extracting, filtering, and labeling relevant tweets for classification.

**Algorithm 1:**
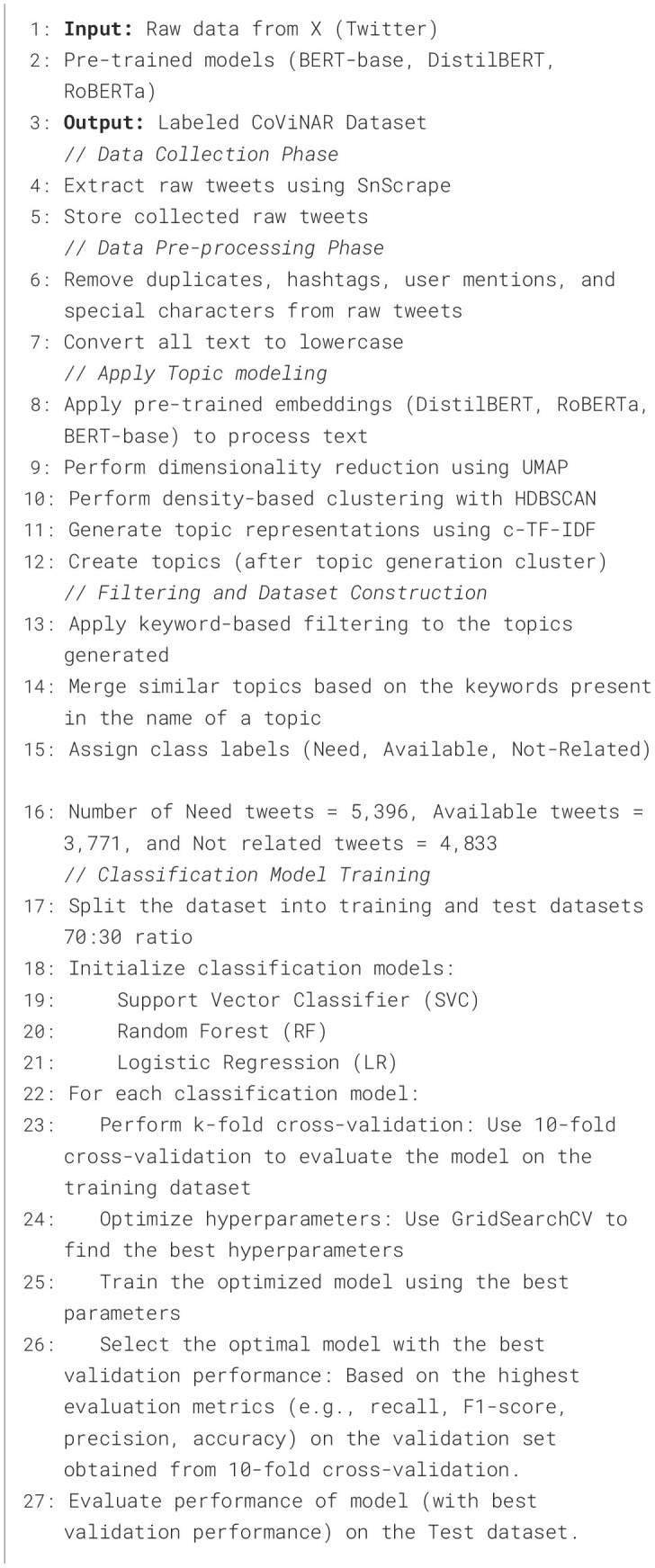
CoViNAR dataset creation and classification framework.

### 4.2 Labeling

To build the targeted CoViNAR dataset, a multi-stage labeling process was used. The initial corpus included about 800,000 pre-processed tweets. First, BERTopic was applied to filter out irrelevant content. This technique used contextual embeddings from the Sentence Transformer model “all-MiniLM-L6-v2” (384-dimensional), UMAP (configured with 20 neighbors, 5 components, and cosine distance) for dimensionality reduction, and HDBSCAN (min cluster size = 21, min samples = 2, Euclidean metric) for clustering. The key parameters and their impact on topic coherence are summarized in Table 2. The optimized BERTopic model identified between 700 and 900 semantically coherent topics, each achieving a coherence score of 0.75 or higher, validating the effectiveness of the selected parameters. The topic representation model was set to “KeyBERTInspired”, and an n-gram range of (1,3) was used to capture unigrams, bigrams, and trigrams, enriching the contextual understanding.

**Table 2:** Example of the relevant topic.

**Topic**	**Count**	**Name**	**Relevance**
23	264	23_australia will suspend_australia suspends_suspends flights from_suspends flights	No
25	255	25_more sarscov2 covid19_sarscov2 covid19_covid19 sarscov2_covid19 sars	No
26	253	26_icu beds availble_delhi covidhlep beds_only icu beds_icu beds	Yes
27	247	27_crisis womenrespond2crises wphfund_womenrespond2crises wphfund_crisis	No
29	237	29_verified delhi oxygen_delhi oxygen_city delhi oxygen_delhi oxygen contact	Yes
36	219	36_oxygen bed urgently_need oxygen bed_required oxygen bed_oxygen bed in	Yes
50	176	50_covid19 india_covid19 india was_indias covid_india us	No

From the initial dataset of 8 M, 114,345 tweets were retained after topic modeling and relevance filtering. Topics were automatically labeled based on keyword-driven topic names, and only those marked as relevant (e.g., ICU bed availability, verified oxygen suppliers) were selected for further analysis. Table 3 provides examples of relevant and irrelevant topics identified by the BERTopic model. Irrelevant topics (e.g., general news or flight suspensions) were excluded. Following this, tweet-level filtering was conducted using keyword and phrase matching. A rule-based approach utilizing regular expressions was applied to identify intent-specific language. This step categorized tweets as follows: Need (14,183 tweets) tweets expressing urgent requirements (e.g., “need oxygen,” “urgently required”); Available (4,809 tweets) tweets indicating available resources (e.g., “beds free at,” “oxygen available”); and Not Relevant (95,353 tweets) tweets discussing COVID-19 but not addressing resource needs or availability.

**Table 3:** Analysis of UMAP, HDBSCAN, and BERTopic parameters on topic coherence.

**Component**	**Key parameters**	**Values tested**	**Best value(s)**	**Impact on topic coherence**
UMAP	n_neighbors	5, 10, 15, 20, 25	20	Lower values led to fragmented topics; higher values caused overly broad clusters. Twenty provided optimal granularity
	min_dist	0.0, 0.1, 0.3, 0.5	0.0	Lower min_dist improved separation of topics; 0.0 helped retain topic diversity
	n_components	2, 3, 5, 10	5	Higher dimensions gave diminishing returns; 5 balanced structure and interpretability
HDBSCAN	min_cluster_size	5, 10, 15, 21, 30	21	Smaller values created noisy micro-clusters; 21 resulted in stable and interpretable topics
	min_samples	1, 2, 5	2	A smaller value reduced noise points while preserving fine-grained topic distinctions
	metric	Euclidean, Cosine, Manhattan	Euclidean	Euclidean performed best with the embedding distribution from MiniLM
Combined	Interaction effects	Tuned jointly	(20, 0.0, 21)	Optimal coherence was achieved when UMAP and HDBSCAN parameters were fine-tuned together
BERTopic	representation_model	Default, KeyBERTInspired	KeyBERTInspired	Helped extract richer topic terms by leveraging contextual embeddings
	n_gram_range	(1,1), (1,2), (1,3)	(1,3)	Including trigrams added contextual richness to topic terms and improved coherence

**Figure 3:**
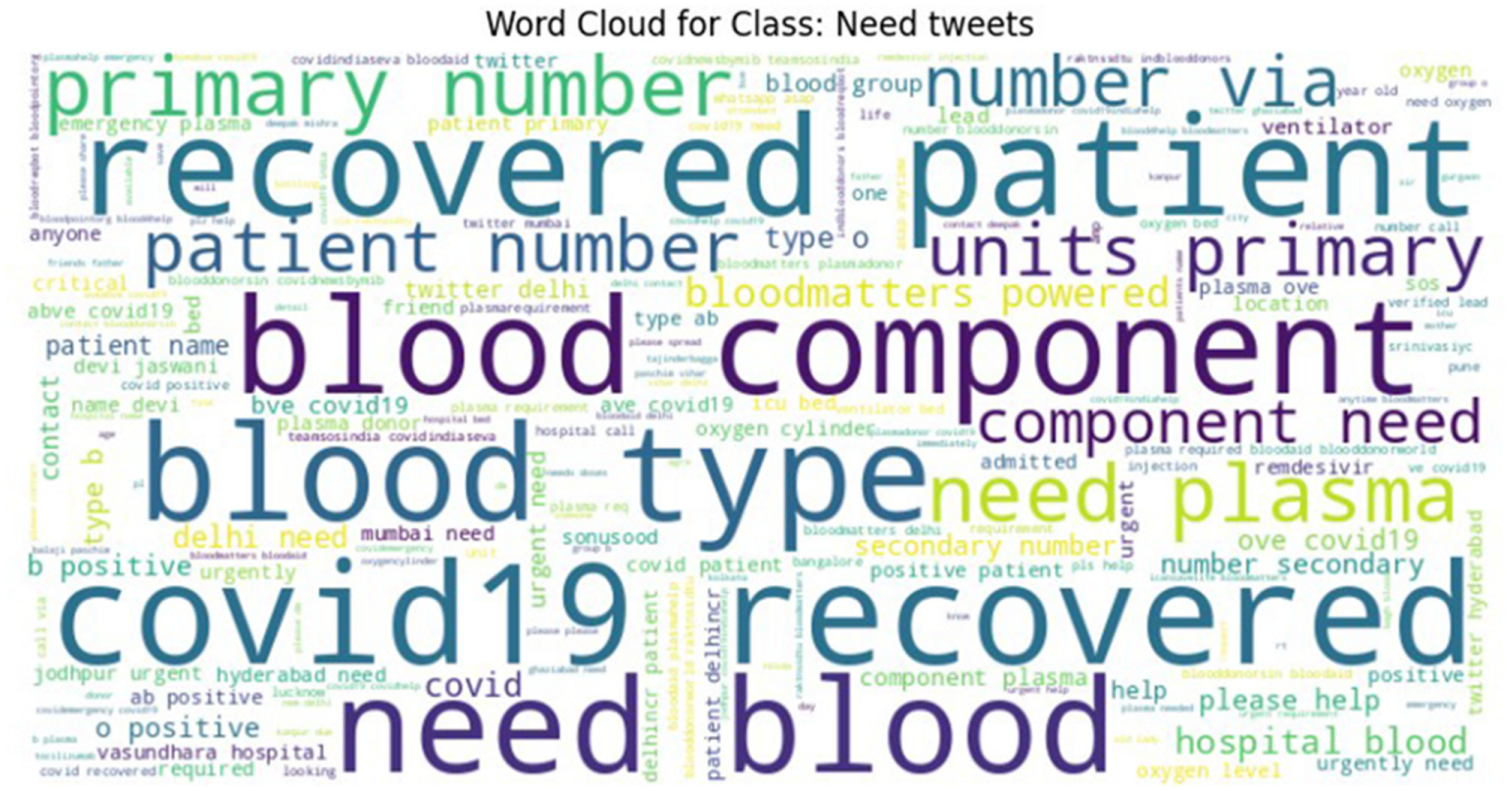
Word cloud representing the most frequent terms in the NEED class of the CoViNAR dataset.

**Figure 4:**
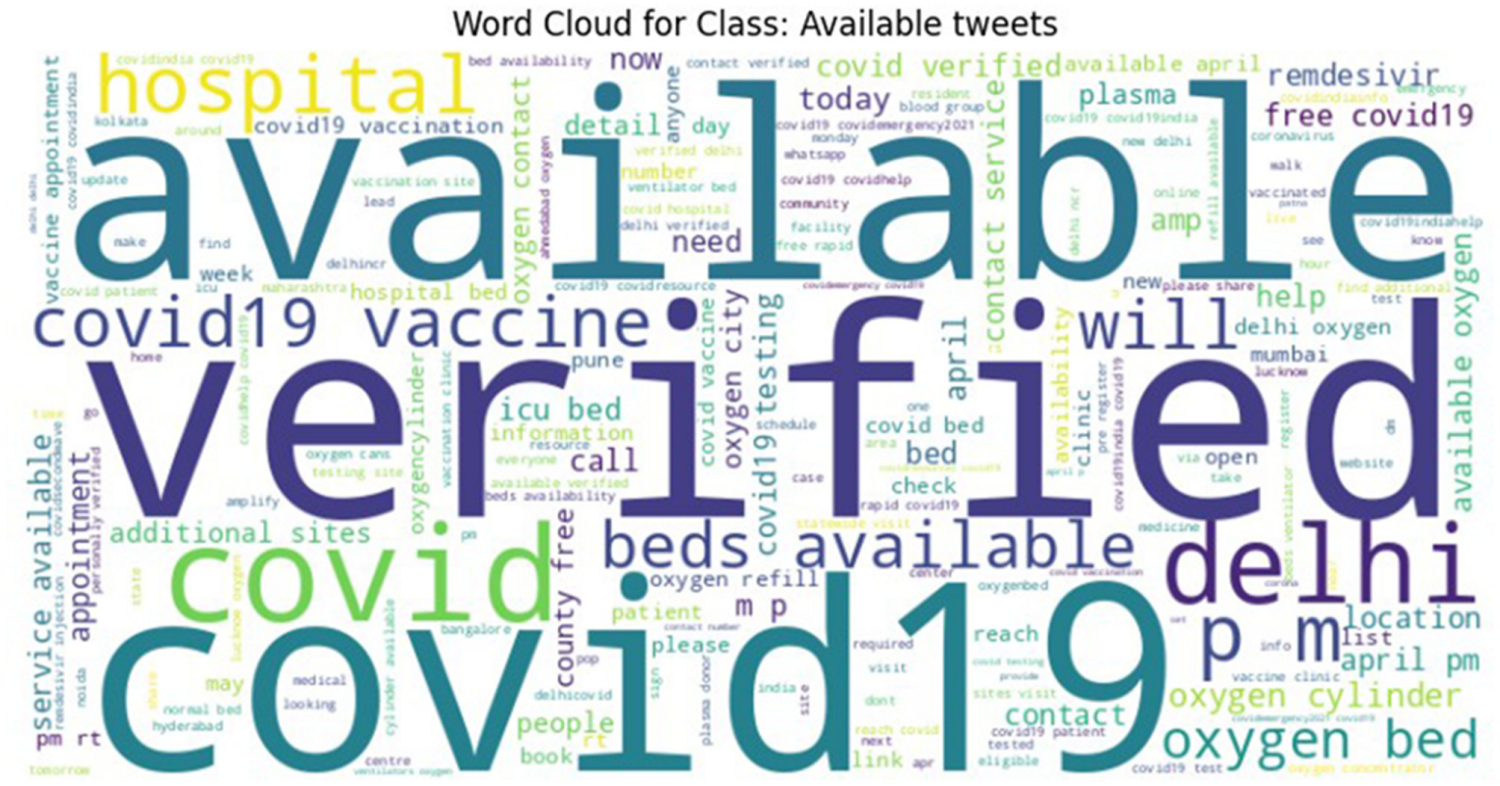
Word cloud representing the most frequent terms in the AVAILABLE class of the CoViNAR dataset.

**Figure 5:**
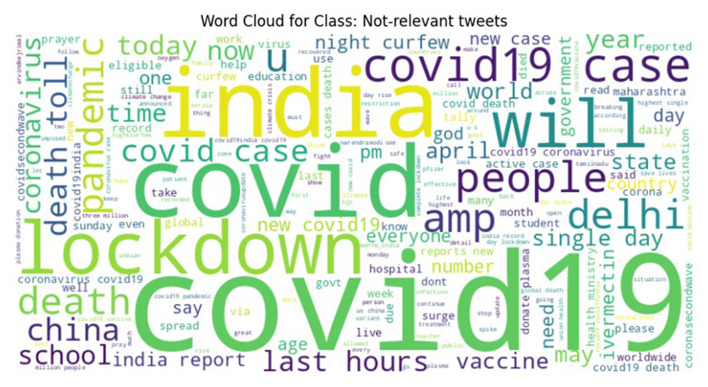
Word cloud representing the most frequent terms in the Not-Relevant class of the CoViNAR dataset.

To ensure high labeling quality, a manual verification step was conducted by human expert (first author) on the tweets labeled as Need, Available, and Not Relevant, refining ambiguous or borderline cases. From this verified subset, a final dataset with near-equal class distribution was curated for the CoViNAR dataset, comprising 5,396 Need, 3,771 Available, and 4,833 Not Relevant tweets. Word clouds were generated to visualize the most frequent terms in each class, as shown in Figures 3–5. These word clouds provide an intuitive representation of the key themes and vocabulary associated with each category of tweets. This final dataset is both semantically coherent and intent-specific, making it well-suited for downstream classification tasks and real-time public health monitoring.

### 4.3 Model design

**Figure 6:**
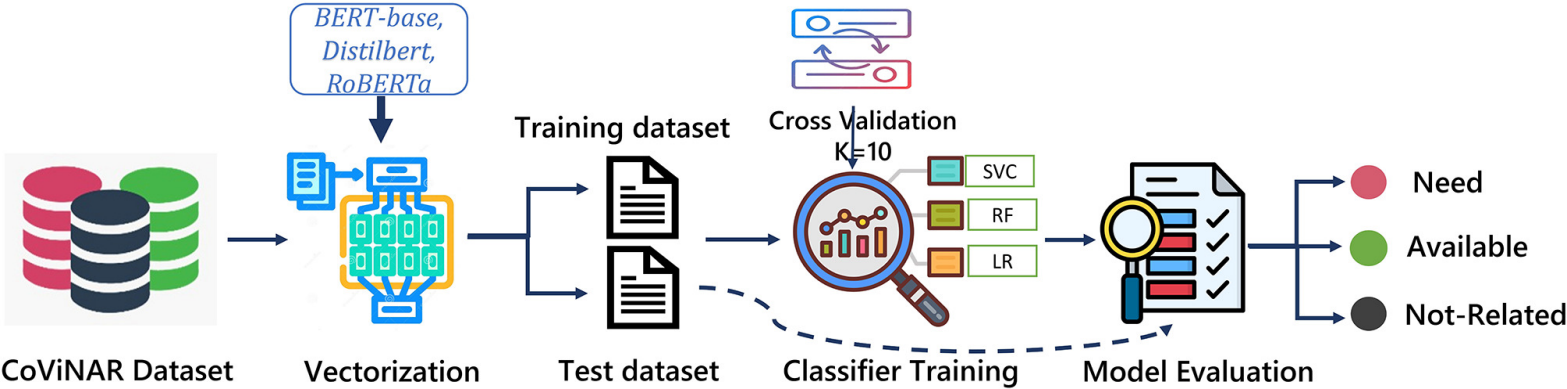
Classification framework for CoViNAR dataset.

This subsection describes the design and development of the NAR classification model trained using the labeled CoViNAR dataset as depicted in Figure 6. The three text embedding based features (detailed in Section 3.3) were experimented with training of multiple classifiers including SVM, RF, and LR, using the CoViNAR dataset. After vectorization, we split the dataset into 70% training and 30% testing subsets, with stratification based on the “label” column to maintain class distribution. The training dataset was further partitioned into 10 folds for incorporating 10-fold cross-validation while training. The complete dataset specifications are provided in Table 4.

**Table 4:** Details of the CoViNAR dataset.

**NAR dataset**	**Need**	**Available**	**Not relevant**
Train tweets	3,777	2,640	3,383
Test tweets	1,619	1,131	1,450
Total tweets	5,396	3,771	4,833

Since the three classes in the dataset were nearly balanced, certain measures were taken to address this issue by including the Scikit-Learn parameter “class weight = balanced” in all classification models. This parameter automatically adjusts the class weights, effectively minimizing dataset imbalances during model training. The performance of the classifiers was examined and compared across the above-discussed text embeddings to discover which vector representation method worked best for the tweet classification task. Metrics like accuracy, recall, precision, and F1-score were taken into consideration to assess both embedding quality and model efficacy. To ascertain generalizability over numerous train test splits, stratified 10-fold cross-validation was applied to each classification model.

### 4.4 Hyperparameter optimization

To find the ideal parameters for every classification model trained on the CoViNAR dataset, popular classifiers like SVM, RF, and LR were fine-tuned using GridSearchCV (Pedregosa et al., 2011) and manual optimization techniques. We assessed various parameters for each model considered, along with the embedding pair. Optimal parameters that produced the best classification accuracy on the test dataset are mentioned in Table 5. We ultimately found that DistilBERT embeddings with an SVM classifier give an accurate classification of tweets with 96.42% accuracy, 96.43% f1-score, 96.44% of precision, and 96.42% recall on the CoViNAR dataset.

**Table 5:** Classification models with optimal parameters and search space.

**Model**	**Vectorization techniques**	**Optimal parameters**	**Search space explored**
SVC	DistilBERT	kernel = poly, C = 10, gamma = scale	C: [0.1, 1, 10]; kernel: [linear, poly, rbf]; degree: [2, 3, 4]; gamma: [scale]; class_weight: [balanced]
	RoBERTa	kernel = linear, C = 10, gamma = scale	
	BERT-base	kernel = poly, C = 10, gamma = scale	
RF	DistilBERT	n_estimators = 1000, criterion = entropy, min_samples_split = 3, max_features = sqrt	n_estimators: [100, 300, 1000]; criterion: [gini, entropy]; min_samples_split: [2, 4, 6]; max_features: [sqrt, log2]; class_weight: [balanced]
	RoBERTa	n_estimators = 1000, criterion = entropy, min_samples_split = 4, max_features = sqrt	
	BERT-base	n_estimators = 1000, criterion = gini, min_samples_split = 2, max_features = sqrt	
LR	DistilBERT	max_iter = 1000, penalty = l2, C = 4, solver = lbfgs	C: [0.01, 0.1, 1, 10]; penalty: [l2]; solver: [lbfgs]; max_iter: [1000, 5000]; class_weight: [balanced]
	RoBERTa	max_iter = 5000, penalty = l2, C = 9, solver = lbfgs	
	BERT-base	max_iter = 5000, penalty = l2, C = 0.8, solver = lbfgs	

Hyperparameters were tuned using GridSearchCV with 10-fold stratified cross-validation (cv=10) and macro-averaged F1-score as the scoring metric (scoring='f1_macro').

**kernel**: Specifies the kernel type to be used in the algorithm. **C**: Regularization parameter. **gamma**: Kernel coefficient. **n_estimators**: Number of decision trees in the forest. **criterion**: Function to measure the quality of a split. **min_samples_split**: Minimum samples required to split an internal node. **max_features**: Number of features to consider when looking for the best split. **max_iter**: Maximum number of iterations taken for the solvers to converge.

## 5 Experimental results

For this work, we experimented with almost all classification models available with the Scikit-Learn library, and found these three machine learning (ML) algorithms; Support Vector Classifier (SVC), Logistic Regression (LR), and Random Forest (RF) performing the best toward the classification of context-aware word embeddings of CoViNAR tweets. To transform pre-processed tweets into numerical representations, three pre-trained transformer-based embeddings- DistilBERT, RoBERTa, and BERT-base were employed. These models were chosen for their ability, particularly in the context of tweets, to capture the semantics of the small texts while balancing computational efficiency. To mitigate overfitting and ensure robust evaluation, a stratified 10-fold cross-validation strategy was implemented.

Hyperparameter tuning was performed during cross-validation using GridsearchCV to identify the optimal parameters on a held-out test set for each classifier. We selected the models and their hyperparameter settings that yielded the best cross-validation performance. Once the best models and their parameters were identified, we used them to classify a separate Test dataset. The comparative results of all classifiers, using their best configurations, are presented in Tables 6, 7. Table 6 reports the average stratified 10-fold cross-validation results, while Table 7 presents the Test dataset performance which was not used to obtain parameters of the model. Given the class imbalance in the dataset, a comprehensive evaluation strategy was employed. Instead of relying solely on accuracy, which can be misleading in imbalanced datasets, precision, recall, and F1-score were prioritized. Aditionally, precision-recall analysis was also conducted, as it is particularly useful in imbalanced classification tasks.

**Table 6:** Average stratified 10-fold cross-validation accuracy (%) of classifiers using different text vectorization methods.

**Text vectorization**	**SVC**	**LR**	**RF**
DistilBERT	96.39	96.14	94.54
RoBERTa	95.68	95.45	93.22
BERT-base	95.57	94.77	91.74

**Table 7:** Performance of classifiers on the test dataset using multiple text vectorization methods.

**Classifier**	**Text vectorization**	**Acc (%)**	**F1 (%)**	**Prec (%)**	**Rec (%)**
SVC	DistilBERT	96.42	96.43	96.44	96.42
	RoBERTa	95.92	95.93	95.94	95.92
	BERT-base	95.51	95.50	95.51	95.50
Random forest	DistilBERT	94.14	94.12	94.14	94.14
	RoBERTa	92.83	92.78	92.93	92.83
	BERT-base	91.92	91.88	91.96	91.92
Logistic regression	DistilBERT	95.92	95.92	95.93	95.92
	RoBERTa	95.33	95.34	95.36	95.33
	BERT-base	94.16	94.17	94.16	94.17

**Figure 7:**
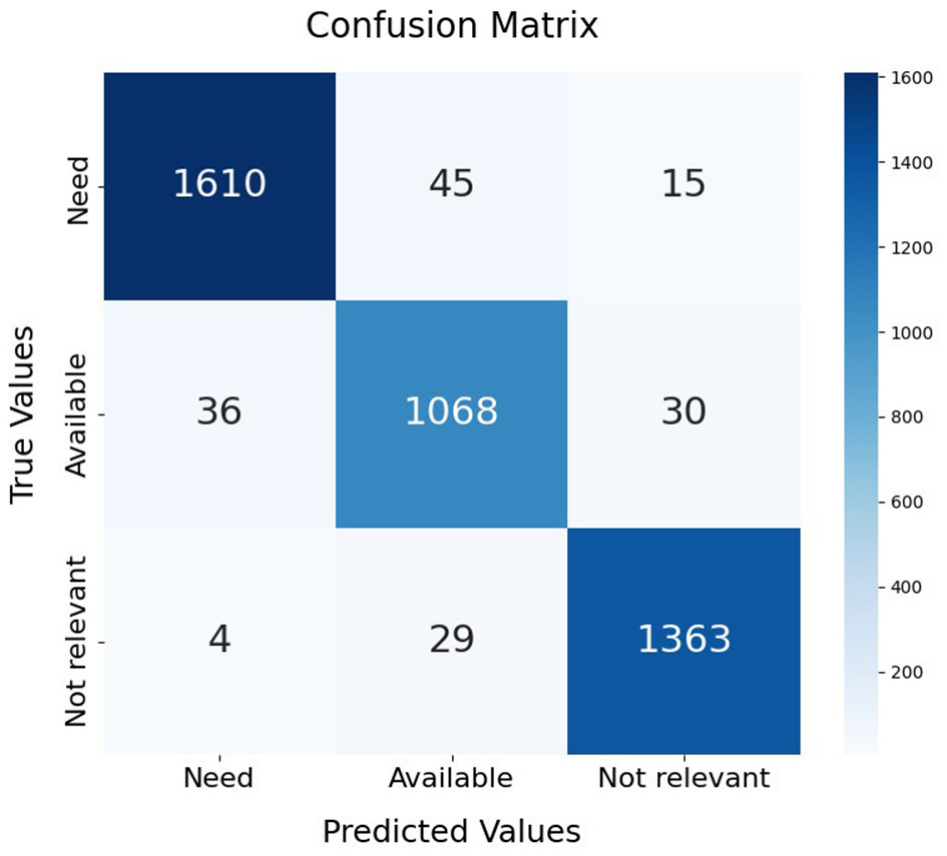
Confusion matrix of support vector classifier trained on the DistilBERT embeddings of CoViNAR dataset.

**Figure 8:**
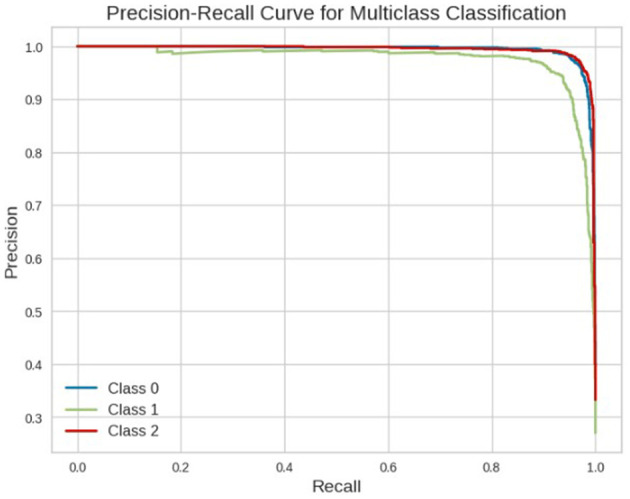
Precision-recall graph for SVC classifier on CoViNAR dataset.

Among the evaluated models, SVC with DistilBERT embeddings achieved the best performance, with an accuracy of 96.42%, F1-score of 96.43%, precision of 96.42%, and recall of 96.42% on the test dataset. The confusion matrix of the best-performing SVC model is visualized in Figure 7. To further validate the classifier's performance on imbalanced data, Figure 8 presents the precision-recall curve, demonstrating its ability to balance false positives and false negatives effectively. In this context, class 0 represents the “not-related” class, class 1 represents the “Available class,” and class 2 represents the “Need” class.

Tweets, being time-sequenced data, were transformed into word embeddings to effectively capture their contextual information. Various classification models were then evaluated, all of which proved suitable for developing a tweet classification system. However, SVM demonstrated consistently superior performance, robustness to high-dimensional embeddings, and better generalization, while maintaining acceptable computational requirements. SVC was the finally selected as NAR classification model because it consistently outperformed Logistic Regression and Random Forest across all tested embeddings over both cross-validation and test datasets. The confusion matrix (Figure 7) demonstrates SVC's strong performance across all classes, with high correct predictions for “Need” (1610), “Available” (1068), and “Not relevant” (1363), and relatively low misclassification rates. While Random Forest and Logistic Regression also achieved good overall results, SVC demonstrated greater stability and higher performance metrics, especially in critical classes like “Need,” which are essential for identifying urgent resource requests. SVC's capacity to separate complex decision boundaries in high-dimensional feature spaces, combined with manageable computational costs, made it the preferred choice for the CoViNAR classifier.

Despite the strong performance of the SVC model, a manual error analysis revealed several misclassified tweets where critical Need cases were incorrectly labeled as Not Related. This is particularly important given that “Need” tweets represent urgent resource requests and misclassifying them could impact real-world response efforts. These errors often involved implicit language, emotional tone, abbreviations, or vague phrasing that the model failed to capture. Table 8 presents a sample of such misclassified tweets along with possible reasons for misclassification. Future work could address these limitations by incorporating context-aware models, better handling of informal or emotional language, and thread-level information where applicable.

**Table 8:** Examples of misclassified tweets with possible reasons.

**Tweet text**	**Actual class**	**Predicted class**	**Reason for misclassification**
In searching of Oxygen. #OxygenCrisis #COVIDEmergency #COVID19	Need	Available	Short and lacks explicit request wording (e.g., “need,” “help”)
Plasma donor in Indore. RT to amplify #PlasmaRequirement	Available	Not related	Lacks verb indicating availability (e.g., “providing”); resembles amplification tweet
Need help getting a vaccine appointment in #Massachusetts? Contact @macovidvaxhelp	Available	Need	Mention of help service confused model into interpreting it as resource offering
Please arrange covid ICU bed for my old mother. #COVID19	Need	Not related	Indirect wording without keywords like “urgent” or “need” may have confused classifier
A person with COVID-19 is seeking a bed and is ready to bear expenses.	Need	Not related	Formal tone and lack of urgency words might have led to misclassification

## 6 Geographic location specific temporal analysis of tweets

Researchers have employed various metrics to assess pandemic severity through sentiment analysis and topic modeling. In this work, we present a temporal analysis of tweets related to the need and availability of resources, offering significant insights into the severity of the pandemic over more than three years. This analysis was performed by mapping the correlation between resource demands expressed on social media and the actual patient counts of COVID-19, as reported by the WHO.

**Figure 9:**
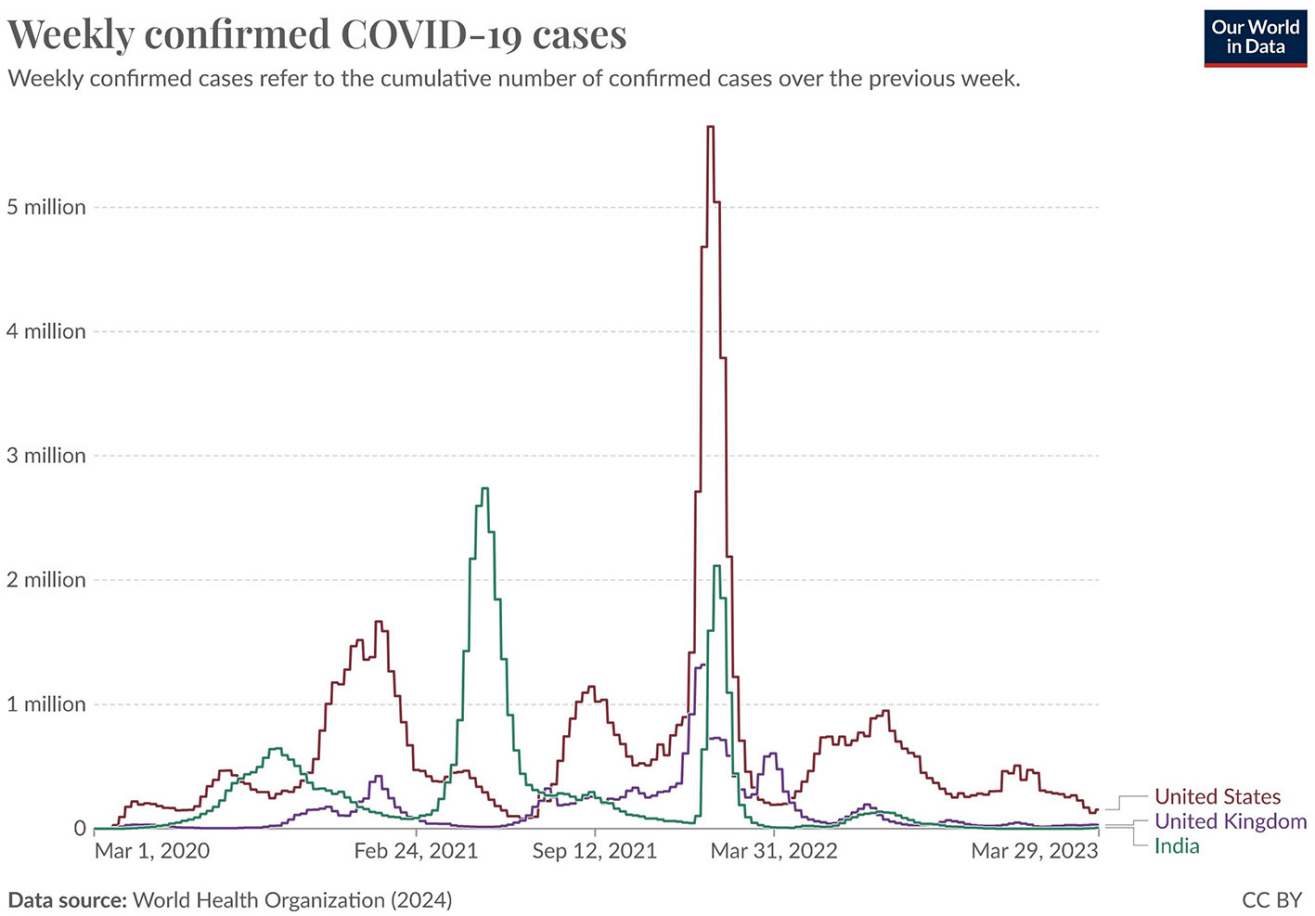
COVID-19 confirmed cases in India, the U.S., and the U.K.

**Figure 10:**
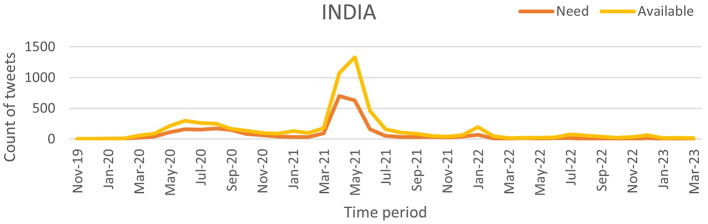
Classified NAR tweets of India.

**Figure 11:**
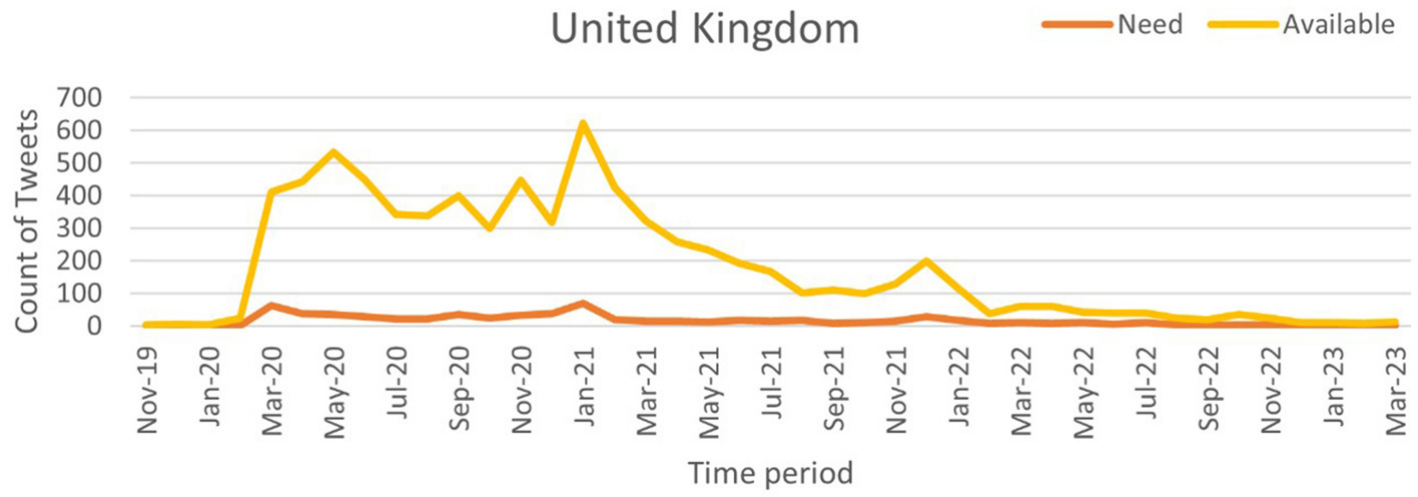
Classified NAR tweets of United Kingdom.

**Figure 12:**
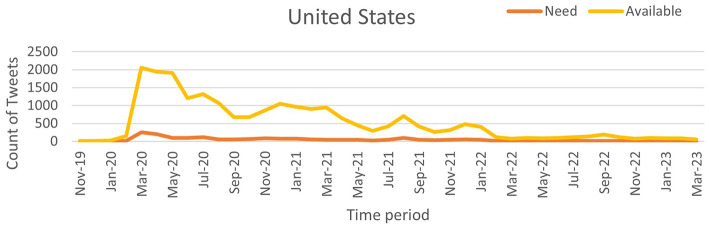
Classified NAR tweets of United States.

Our work utilizes time-series tweet data from November 2019 to March 2023, considering only tweets with location information. These tweets were pre-processed using the proposed NLP pipeline and then classified using the NAR (Need and Available Resources) classifier to analyze pandemic severity across three of the most affected countries: India, the United Kingdom (UK), and the United States (US). The daily counts of “Need” and “Available” tweets were recorded to assess the correlation between social media activity and pandemic severity. Figure 9 (source: Our World in Data) illustrates the weekly confirmed COVID-19 cases for India, the UK, and the US, while Figures 10–12 show the temporal patterns of NAR-classified tweets for these countries. Table 9 provides a comparative summary of NAR tweets across the three nations.

**Table 9:** Comparison of peak “Need” and “Available” tweets across three countries during COVID-19.

**Country**	**Key period**	**“Need” tweets (peak)**	**“Available” tweets (peak)**	**Observations**
India	March 2020	~1,200 tweets per week	< 500 tweets per week	Spike in “Need” tweets during the first COVID-19 wave, indicating resource shortages
	March–July 2021	~1,200 tweets per week	< 500 tweets per week	A sharp increase in “Need” tweets during the second wave, aligning with 400,000+ daily cases and oxygen shortages
United States	January 2021	~400 tweets per week	~800 tweets per week	“Available” tweets consistently outnumbered “Need” tweets, suggesting better resource availability
United Kingdom	January 2021	~300 tweets per week	~600 tweets per week	Similar to the US, “Available” tweets exceeded “Need” tweets, reflecting effective resource management

To quantify the relationship between tweet activity and case counts, we employed the Truncated Time Shift (TTS) test, a strong, non-parametric method for time series correlation. The results showed a strong and significant correlation for India (*r* = 0.9480, *p* = 0.0000) and the UK (*r* = 0.7280, *p* = 0.0000). This confirms that increases in resource-related tweets closely matched pandemic severity. While the US also showed a positive correlation (*r* = 0.4212), it was not significant at the 0.05 level (*p* = 0.0800). This may be due to better baseline resource availability and fewer distress signals on social media. These findings suggest that social media can act as an additional source of information to official surveillance, particularly in areas with limited health infrastructure.

A sharp spike in “Need” tweets was observed in India during March 2020, coinciding with the country's first COVID-19 wave. More notably, between March and June 2021, a significant increase in both “Need” and “Available” tweets aligned with the second wave, which led to nationwide lockdowns and oxygen shortages. In contrast, the US and the UK exhibited a higher frequency of “Available” tweets than “Need” tweets, suggesting better resource availability during the pandemic. This trend indicates that these countries had sufficient resources accessible to the public. Overall, this pattern suggests that during public health emergencies, social media activity can serve as a valuable tool for identifying localized resource shortages and distress. Platforms like X and others may provide health officials with early warnings, enabling them to allocate resources and support to the most affected communities more effectively.

The present work demonstrates the usefulness of the NLP pipeline, which can be applied to unstructured social media data to analyze pandemic severity level analysis and improve preparedness and overall management during an ongoing pandemic. Thus, the proposed NAR classifier utilizes social media data to gain valuable insights into public perception of the ongoing pandemic situation and its perceived severity.

## 7 Conclusion

This work explored the role of social media in pandemic response, focusing on real-time resource management, assessing pandemic severity, and comparing its effectiveness with traditional surveillance methods. Our findings confirm that social media analysis can provide valuable real-time insights, enhancing crisis response strategies.

Addressing RQ1, our results demonstrate that social media data can be effectively utilized for real-time resource management during a pandemic. The CoViNAR dataset and NLP pipeline efficiently identify and classify resource-related tweets, enabling timely responses to emerging needs during crises. By utilizing BERTopic for topic modeling, we improved contextual understanding beyond simple keyword-based filtering, making the process more scalable and adaptable to different crises. For RQ2, the temporal analysis of classified tweets showed strong correlations with real-world COVID-19 case trends, reinforcing the potential of social media analysis as an early-warning mechanism. These findings highlight how social media data can enhance real-time assessments of pandemic severity, offering health professionals actionable insights to anticipate surges and optimize response efforts. Such insights are valuable for real-world public health operations, as they could help authorities identify emerging shortages of critical resources, direct supplies where most urgently needed, and support faster decision-making during health crises. Regarding RQ3, our findings suggest that social media-based monitoring offers timely and granular signals that may be valuable in supplementing traditional resource surveillance systems, particularly during high-demand periods. The ability to classify tweets using machine learning models; particularly the SVM model with DistilBERT embeddings, which achieved 96.42% accuracy and a 96.43% F1 score underscores the effectiveness of NLP-driven approaches in extracting critical information from large-scale social media data.

Despite these promising outcomes, we acknowledge certain limitations. Our reliance on X (formerly Twitter) and English-language data may limit the generalizability of our approach, as social media use and languages vary widely across regions. Future work should expand the dataset to other major languages and platforms and explore domain adaptation techniques to transfer knowledge across languages. Additionally, the proposed methodology is also adaptable to other types of crises, including natural disasters and humanitarian emergencies, where real-time detection of resource needs can greatly support response planning and intervention efforts.

In conclusion, our research highlights the transformative potential of social media analysis in pandemic response, demonstrating its role in real-time decision-making, resource allocation, and crisis preparedness. By addressing the identified limitations and expanding this approach to other health emergencies, social media-driven monitoring can become an invaluable tool for public health professionals, strengthening global resilience against future outbreaks.

## Data Availability

The code and data associated with this work are available on GitHub and can be accessed through the repository via the following link: https://github.com/Sufishafia/CoViNAR/. This repository contains all the necessary files to reproduce the results and implement the methods described in this paper.

## References

[B1] Ahmad Wani M. ELAffendi M. A. Shakil K. A. Shariq Imran A. Abd El-Latif A. A. (2023). Depression screening in humans with ai and deep learning techniques. IEEE Trans. Comput. Soc. Syst. 10, 2074–2089. 10.1109/TCSS.2022.3200213

[B2] Allaoui M. Kherfi M. L. Cheriet A. (2020). “Considerably improving clustering algorithms using umap dimensionality reduction technique: a comparative study,” in International Conference on Image and Signal Processing (Springer: New York), 317–325. 10.1007/978-3-030-51935-3_34

[B3] Bird S. (2006). “Nltk: the natural language toolkit,” in Proceedings of the COLING/ACL 2006 Interactive Presentation Sessions (Sydney, NSW: Association for Computational Linguistics), 69–72. 10.3115/1225403.1225421

[B4] Borges D. Nascimento M. C. (2022). COVID-19 ICU demand forecasting: a two-stage prophet-lstm approach. Appl. Soft Comput. 125:109181. 10.1016/j.asoc.2022.109181 35755299 PMC9212961

[B5] Breiman L. (2001). Random forests. Mach. Learn. 45, 5–32. 10.1023/A:101093340432440797221

[B6] Cortes C. Vapnik V. (1995). Support-vector networks. Mach. Learn. 20, 273–297. 10.1023/A:102262741141140797221

[B7] Da T. Yang L. (2020). Local COVID-19 severity and social media responses: evidence from china. IEEE Access 8, 204684–204694. 10.1109/ACCESS.2020.3037248 34786296 PMC8545260

[B8] Devlin J. Chang M.-W. Lee K. Toutanova K. (2019). “Bert: pre-training of deep bidirectional transformers for language understanding,” in Proceedings of the 2019 Conference of the North American Chapter of the Association for Computational Linguistics: Human Language Technologies, Volume 1 (Long and Short Papers) (Minneapolis, MN: Association for Computational Linguistics), 4171–4186.

[B9] Egger R. Yu J. (2022). A topic modeling comparison between lda, nmf, top2vec, and bertopic to demystify twitter posts. Front. Sociol. 7:886498. 10.3389/fsoc.2022.886498 35602001 PMC9120935

[B10] Goic M. Bozanic-Leal M. S. Badal M. Basso L. J. (2021). COVID-19: short-term forecast of ICU beds in times of crisis. PLoS ONE 16:e0245272. 10.1371/journal.pone.0245272 33439917 PMC7806165

[B11] Grootendorst M. (2022). Bertopic: neural topic modeling with a class-based tf-idf procedure. arXiv preprint arXiv:2203.05794. 10.48550/arXiv.2203.05794

[B12] Gupta A. Katarya R. (2020). Social media based surveillance systems for healthcare using machine learning: a systematic review. J. Biomed. Inform. 108:103500. 10.1016/j.jbi.2020.103500 32622833 PMC7331523

[B13] Hosmer Jr D. W. Lemeshow S. Sturdivant R. X. (2013). Applied Logistic Regression. Hoboken, NJ: John Wiley & Sons. 10.1002/9781118548387

[B14] Jalil Z. Abbasi A. Javed A. R. Badruddin Khan M. Abul Hasanat M. H. Malik K. M.. (2022). COVID-19 related sentiment analysis using state-of-the-art machine learning and deep learning techniques. Front. Public Health 9:812735. 10.3389/fpubh.2021.812735 35096755 PMC8795663

[B15] Jatnika D. Bijaksana M. A. Suryani A. A. (2019). Word2vec model analysis for semantic similarities in english words. Procedia Comput. Sci. 157, 160–167. 10.1016/j.procs.2019.08.153

[B16] Kaur H. Ahsaan S. U. Alankar B. Chang V. (2021). A proposed sentiment analysis deep learning algorithm for analyzing COVID-19 tweets. Inf. Syst. Front. 23, 1417–1429. 10.1007/s10796-021-10135-7 33897274 PMC8057010

[B17] Kaur M. Cargill T. Hui K. Vu M. Bragazzi N. L. Kong J. D. (2024). A novel approach for the early detection of medical resource demand surges during health care emergencies: infodemiology study of tweets. JMIR Form. Res. 8:e46087. 10.2196/46087 38285495 PMC10862249

[B18] Lamsal R. Harwood A. Read M. R. (2022). Twitter conversations predict the daily confirmed COVID-19 cases. Appl. Soft Comput. 129:109603. 10.1016/j.asoc.2022.109603 36092470 PMC9444159

[B19] Li C. Chen L. J. Chen X. Zhang M. Pang C. P. Chen H. (2020). Retrospective analysis of the possibility of predicting the COVID-19 outbreak from internet searches and social media data, China, 2020. Eurosurveillance 25:2000199. 10.2807/1560-7917.ES.2020.25.10.2000199 32183935 PMC7078825

[B20] Liu Y. Ott M. Goyal N. Du J. Joshi M. Chen D.. (2019). Roberta: a robustly optimized bert pretraining approach. arXiv preprint arXiv:1907.11692. 10.48550/arXiv.1907.11692

[B21] Maharana K. Mondal S. Nemade B. (2022). A review: data pre-processing and data augmentation techniques. Glob. Transit. Proc. 3, 91–99. 10.1016/j.gltp.2022.04.020

[B22] Marie-Sainte S. L. Alalyani N. Alotaibi S. Ghouzali S. Abunadi I. (2018). Arabic natural language processing and machine learning-based systems. IEEE Access 7, 7011–7020. 10.1109/ACCESS.2018.2890076

[B23] McInnes L. Healy J. Astels S. (2017). hdbscan: hierarchical density based clustering. J. Open Source Softw. 2:205. 10.21105/joss.00205

[B24] McInnes L. Healy J. Melville J. (2018). Umap: uniform manifold approximation and projection for dimension reduction. arXiv preprint arXiv:1802.03426. 10.21105/joss.00861

[B25] McMahon D. E. Peters G. A. Ivers L. C. Freeman E. E. (2020). Global resource shortages during COVID-19: bad news for low-income countries. PLoS Negl. Trop. Dis. 14:e0008412. 10.1371/journal.pntd.0008412 32628664 PMC7337278

[B26] Medford R. J. Saleh S. N. Sumarsono A. Perl T. M. Lehmann C. U. (2020). An “infodemic”: leveraging high-volume twitter data to understand early public sentiment for the coronavirus disease 2019 outbreak. Open Forum Infect. Dis. 7:ofaa258. 10.1093/ofid/ofaa258 33117854 PMC7337776

[B27] Melton C. A. White B. M. Davis R. L. Bednarczyk R. A. Shaban-Nejad A. (2022). Fine-tuned sentiment analysis of COVID-19 vaccine-related social media data: comparative study. J. Med. Internet Res. 24:e40408. 10.2196/40408 36174192 PMC9578521

[B28] Nelson S. P. Raja R. Eswaran P. Alzabut J. Rajchakit G. (2024). Modeling the dynamics of COVID-19 in Japan: employing data-driven deep learning approach. Int. J. Mach. Learn. Cybern. 1–14. 10.1007/s13042-024-02301-5

[B29] Nemes L. Kiss A. (2021). Social media sentiment analysis based on COVID-19. J. Inf. Telecommun. 5, 1–15. 10.1080/24751839.2020.1790793

[B30] O'Leary D. E. Storey V. C. (2020). A google-wikipedia-twitter model as a leading indicator of the numbers of coronavirus deaths. Intell. Syst. Account. Finance Manag. 27, 151–158. 10.1002/isaf.1482

[B31] Pastor C. K. (2020). Sentiment analysis of filipinos and effects of extreme community quarantine due to coronavirus (COVID-19) pandemic. SSRN Electron. J. 10.2139/ssrn.3574385

[B32] Pedregosa F. Varoquaux G. Gramfort A. Michel V. Thirion B. Grisel O.. (2011). Scikit-learn: machine learning in python. J. Mach. Learn. Res. 12, 2825–2830. 10.5555/1953048.207819534820480

[B33] Pennington J. Socher R. Manning C. D. (2014). “Glove: global vectors for word representation,” in Proceedings of the 2014 Conference on Empirical Methods in Natural Language Processing (EMNLP) (Doha: Association for Computational Linguistics), 1532–1543. 10.3115/v1/D14-1162

[B34] Qin L. Sun Q. Wang Y. Wu K.-F. Chen M. Shia B.-C.. (2020). Prediction of number of cases of 2019 novel coronavirus (COVID-19) using social media search index. Int. J. Environ. Res. Public Health 17:2365. 10.3390/ijerph17072365 32244425 PMC7177617

[B35] Ranney M. L. Griffeth V. Jha A. K. (2020). Critical supply shortages—the need for ventilators and personal protective equipment during the COVID-19 pandemic. N. Engl. J. Med. 382:e41. 10.1056/NEJMp2006141 32212516

[B36] Reimers N. Gurevych I. (2019). Sentence-bert: sentence embeddings using siamese bert-networks. arXiv preprint arXiv:1908.10084. 10.18653/v1/D19-141036568019

[B37] Roman M. Shahid A. Khan S. Koubaa A. Yu L. (2021). Citation intent classification using word embedding. IEEE Access 9, 9982–9995. 10.1109/ACCESS.2021.305054740127378

[B38] Sanh V. Debut L. Chaumond J. Wolf T. (2019). Distilbert, a distilled version of bert: smaller, faster, cheaper and lighter. arXiv preprint arXiv:1910.01108. 10.48550/arXiv.1910.0110839588066

[B39] Shafiya S. Jabin S. (2023). “Current trends in social-media based disease outbreak prediction and surveillance systems,” in 2023 International Conference on Recent Advances in Electrical, Electronics and Digital Healthcare Technologies (REEDCON) (IEEE), 205–210. 10.1109/REEDCON57544.2023.10151369

[B40] Srinivas G. Maanasa R. Meenakshi M. Adaikalam J. Seshayyan S. Muthuvel T. (2021). Ethical rationing of healthcare resources during COVID-19 outbreak. Ethics Med. Public Health 16:100633. 10.1016/j.jemep.2021.100633 33585668 PMC7869626

[B41] Syrowatka A. Kuznetsova M. Alsubai A. Beckman A. L. Bain P. A. Craig K. J. T.. (2021). Leveraging artificial intelligence for pandemic preparedness and response: a scoping review to identify key use cases. NPJ Digit. Med. 4:96. 10.1038/s41746-021-00459-8 34112939 PMC8192906

[B42] Verma H. Mandal S. Gupta A. (2022). Temporal deep learning architecture for prediction of COVID-19 cases in india. Expert Syst. Appl. 195:116611. 10.1016/j.eswa.2022.116611 35153389 PMC8817764

[B43] Wang A. Dara R. Yousefinaghani S. Maier E. Sharif S. (2023). A review of social media data utilization for the prediction of disease outbreaks and understanding public perception. Big Data Cogn. Comput. 7:72. 10.3390/bdcc7020072

[B44] Wani M. A. Bours P. Agarwal N. Jabin S. (2019). “Emotion-based mining for gender prediction in online social networks,” in Proceedings of the ACM, International Conference on Machine Learning and Data Science (Hyderabad: ACM (Association for Computing Machinery)).

[B45] Wani M. A. ELAffendi M. Shakil K. A. Abuhaimed I. M. Nayyar A. Hussain A.. (2024). Toxic fake news detection and classification for combating COVID-19 misinformation. IEEE Trans. Comput. Soc. Syst. 11, 5101–5118. 10.1109/TCSS.2023.3276764

[B46] Wilson A. E. Lehmann C. U. Saleh S. N. Hanna J. Medford R. J. (2021). Social media: a new tool for outbreak surveillance. Antimicrob. Stewardsh. Healthc. Epidemiol. 1:e50. 10.1017/ash.2021.225 36168466 PMC9495414

[B47] Zhuang Z. Cao P. Zhao S. Han L. He D. Yang L. (2021). The shortage of hospital beds for COVID-19 and non-COVID-19 patients during the lockdown of Wuhan, China. Ann. Transl. Med. 9:200. 10.21037/atm-20-5248 33708827 PMC7940947

